# Adaptation of *Graesiella emersonii* Strains to Atmospheric and Enriched CO_2_: Exploring Growth and Photosynthetic Efficiency

**DOI:** 10.3390/bioengineering12101061

**Published:** 2025-09-30

**Authors:** Dora Allegra Carbone, Nicola D’ambrosio, Antonino Pollio

**Affiliations:** 1 Laboratory of Biological Oceanography, Stazione Zoologica “A. Dohrn”, Villa Comunale, 80121 Naples, Italy; 2 Department of Electrical Engineering and Information Technology, Università di Napoli Federico II, Via Claudio 21, 80125 Napoli, Italy; 3 Department of Biology, Università di Napoli Federico II, Via Vicinale Cupa Cinthia 26, 80126 Napoli, Italy

**Keywords:** photosynthetic performance, CO_2_ enrichment, microalgal biotechnology, Aeroterrestrial microalgae

## Abstract

*Graesiella emersonii*, an aeroterrestrial green microalga, exhibits high adaptability to extreme environmental conditions, making it of interest for biotechnological applications. Investigating photosynthetic performance is essential to select high-yield strains and optimize the sustainable production of biomass and bio-products. In this study, two strains (053 and 054) were cultured under atmospheric (0.04%) and elevated (2%) CO_2_ conditions to analyze growth, pigment content, and photosynthesis. Strain 053 showed superior photosynthetic performance and productivity under atmospheric conditions, whereas 2% CO_2_ enhanced growth in both strains, with a significant increase in photosynthetic efficiency in strain 054. The observed differences highlight strain-specific adaptations to CO_2_ availability and suggest the potential of each strain depending on the cultivation environment.

## 1. Introduction

Aeroterrestrial unicellular microalgae of the genus *Graesiella* represent a promising resource for biotechnological applications, including biofuel production, the synthesis of bioactive compounds, and biofiltration systems [1,2,3]. Their remarkable tolerance to extreme environmental conditions makes them suitable candidates for diverse industrial processes [4,5]. Despite their growing relevance, there is still limited understanding of the physiological mechanisms that regulate their growth and photosynthetic activity under variable CO_2_ levels, particularly in industrially relevant settings [6]. Moreover, the differences in physiological responses between strains of the same species originating from distinct ecological environments remain largely unexplored [7,8].

The genus *Graesiella* has attracted increasing attention due to its capacity to produce high-value compounds, such as carotenoids and fatty acids, especially under elevated CO_2_ conditions [9,10]. For instance, *Graesiella emersonii* WBG-1 has been shown to accumulate up to 66% protein under heterotrophic cultivation, setting it apart from other microalgal species [11]. Nevertheless, the response of this species to fluctuating CO_2_ levels is still poorly characterized, limiting its optimization for industrial-scale applications [12,13,14].

To address this gap, the present study compares two distinct *Graesiella emersonii* strains (053 and 054, ACUF collection) isolated from different ecological niches. Their photosynthetic efficiency and growth were evaluated under two CO_2_ regimes: atmospheric and enriched to 2%. The investigation included classical growth and pigment analyses alongside a suite of photochemical and gas exchange measurements, offering a comprehensive assessment of their physiological behavior.

Understanding these strain-specific differences is crucial for determining their productivity under controlled CO_2_ conditions, which is fundamental for industrial applications. Microalgae-based carbon capture and utilization (CCUS) technologies benefit directly from such insights, as selecting high-performing strains can enhance photosynthetic efficiency, metabolite production, and biomass yield, thereby improving the overall performance of bioreactors [15,16,17]. Ultimately, these findings can support the development of sustainable industrial processes and contribute to the production of environmentally friendly, high-value biomass for diverse biotechnological applications.

## 2. Material and Methods

### 2.1. Strains

The algal strains 053 and 054 used in this study were obtained from the Algal Collection of the Department of Biology, University of Naples “Federico II” (ACUF; available at www.acuf.net). The strains were initially identified as *Chlorella emersonii* by Shihira and Kraus [18] both strains are collected in Pennsylvania (USA). 053 was collected on bark while 054 was collected from fresh water. It was later reassigned to the genus *Scenedesmus*, being classified as *Scenedesmus vacuolatus* by Kessler and collaborators in 1997. A further taxonomic revision carried out by Nozaki, Katagiri, Nakagawa, Aizawa, and Watanabe in 2017 led to its current designation as *Graesiella emersonii* [19].

### 2.2. Culture Medium

Algal cultures were maintained in modified Bold’s Basal Medium (BBM) [8] enriched with 40 mg L^−1^ of sodium nitrate (NaNO_3_) as the nitrogen source. The medium was sterilized by autoclaving at 120 °C for 20 min. Following sterilization, the pH was approximately 7.0.

### 2.3. Photobioreactor Design and Operating Conditions

Microalgae were cultured in vertical bubble column reactors (VBC), as described by Olivieri et al. [8] (additional information in Supplementary Materials S1). The VBC consisted of a 1 L cylindrical vessel with an operating volume of 600 mL. The light intensity was set to 250 μmol photons m^−2^ s^−1^ warm-white LED light under atmospheric CO_2_ conditions (approximately 0.04% CO_2_ by volume, or 400 ppm) and 2% CO_2_ (20,000 ppm), with air sparging. To prevent excessive cell accumulation at the surface, regular bubbling was employed to promote mixing within the photobioreactor, ensuring a homogeneous culture environment.

The experiments were conducted under batch culture conditions, meaning that the cultures were grown without further addition of nutrients or fresh medium after the initial inoculation. This approach allows for the observation of growth dynamics in a closed system for approximately six days. In this system, the final culture volume was maintained constant by accounting for water loss due to sampling and evaporation from the gas bubbles.

### 2.4. pH Measurement

The pH was determined using a benchtop pH meter (Mettler Toledo, Columbus, OH, USA).

### 2.5. Analysis of Growth

#### 2.5.1. Cell Counting

1 mL of the culture was collected daily throughout the duration of the experiment for cell count measurements. The cell counts were performed using a Bürker chamber. A small volume of the sample was mixed with a fixed quantity of water, and a few drops of the mixture were then placed onto the Bürker chamber grid. The number of cells in the designated grid area was counted under a microscope (using a 400× magnification). Cell density was calculated by counting the cells in multiple grid areas, with measurements taken in triplicate at the end of the experiment to ensure accuracy.

#### 2.5.2. Determination of Biomass

In liquid culture systems, 2 mL of the culture was collected every two days over a period of six days starting from biomass levels of 0.3 g L^−1^, in triplicate, using a sterile syringe for dry weight determination. The samples were filtered onto polycarbonate membranes using a vacuum pump, then lyophilized for two hours using a freeze dryer. Final dry weights were measured with an analytical balance (Sartorius, Bovenden, Germany).

#### 2.5.3. Growth Rate

The growth rate **μ** (d^−1^) over the experimental period was determined using the following formula:

(1) $$\mu =\frac{\ln X_{t\;}-lnX_{0}}{t}$$

**μ** = Growth rate (in days^−1^ or h^−1^, depending on the time unit used).

**Xt** = Biomass concentration at time **t** (g L^−1^ or cells/mL).

**X0** = Initial biomass concentration (g L^−1^ or cells/mL).

**t** = Elapsed time, which can be expressed in days or hours (depending on the time scale used).

### 2.6. Photosynthetic Performance Assessment

#### 2.6.1. Photochemical Activity

The photochemical activity of the two strains was evaluated using a Pulse Amplitude Modulation (PAM) fluorometry at ambient temperature in a thermostatic chamber. Measurements were conducted with a pulse-modulated fluorometer (FMS-2, Hansatech Instruments Ltd., Pentney, UK). After a dark acclimation period of 30 min, 2 mL of each sample was placed into a quartz cuvette near the optical fiber of the fluorometer, with continuous stirring. The microalgae were exposed to four distinct light intensities: 39 μmol photons m^−2^ s^−1^, 89 μmol photons m^−2^ s^−1^, 300 μmol photons m^−2^ s^−1^, and 496 μmol photons m^−2^ s^−1^. A cycle of 3 min of light and 3 min of darkness was used [12].

The following parameters were measured [20,21].

- Maximum Efficiency of Photosystem II (F*_v_*/F*_m_*):

This parameter reflects the maximum efficiency of photosystem II (PSII). F_v_/F_m_ is determined in the dark using a saturating light pulse of 2500 μmol photons m^−2^ s^−1^ for 0.6 s. The formula is:

(2) $$\frac{F_{v}}{F_{m}}=\frac{F_{m\;\;}-F_{0}}{F_{0}}$$

where

○ F_m_ = Maximum fluorescence intensity of the culture in the dark-adapted state applying a saturating light pulse
○ F_o_ = Minimum fluorescence intensity (dark-adapted state)

- *Non-Photochemical Quenching (NPQ):*

NPQ measures the dissipation of excess light energy as heat within PSII. NPQ is calculated according to the Stern-Wolmer equation [22].

(3) $$NPQ=\frac{F_{m}-{F^{\prime }}_{m}}{{F^{\prime }}_{m}}$$

○ **F_m_** = Maximum fluorescence in the dark-adapted state (typically measured after a strong light pulse).
○ F’_m_ = Maximum fluorescence at the light adapted light (not at saturation), i.e., the maximum fluorescence measured when the culture is exposed to growth light (not in saturation).

The quantum yield of PSII (ΦPSII)

This parameter indicates the photochemical efficiency of PSII under illumination. It is a direct measure of the ability of PSII to convert absorbed light into chemical energy. It is calculated as the ratio of variable fluorescence (F_v_) to the maximum fluorescence (F_m_) during exposure to light. The formula for ΦPSII [23] is:

(4) $$\Phi PSII=\frac{{F^{\prime }}_{m}-F^{\prime }}{{F^{\prime }}_{m\;\;\;\;\;}}$$

**F’ₘ** = Maximum fluorescence under light conditions (when the culture is exposed to growth light).

**F’** = Steady-state fluorescence under light conditions (when the culture is exposed to growth light, in non-saturating light).

#### 2.6.2. Chlorophyll a (Chla) Content

The in vivo chlorophyll a concentration was measured using a handheld fluorometer (AquaFluorTM; Turner Designs, San Jose, CA, USA), which also serves as a turbidimeter. The OD readings provided by the instrument were correlated with biomass concentration through calibration against spectrophotometric measurements at 750 nm.

#### 2.6.3. Gas Exchange

Oxygen evolution in the samples was assessed using an oxygraph (Oxygraph Hansatech), equipped with a thermostatic control set to 24 °C and S1 Clark-type oxygen electrodes. To prevent light limitation during the experiments, the biomass concentration was consistently maintained at 0.12 g L^−1^ for all gas exchange measurements. Four light intensities (photosynthetic photon flux rate, PFR) ranging from 39, 80, 290, and 500 μmol photons m^−2^ s^−1^ were applied. The oxygen control unit allowed for the simultaneous measurement of the following:

- P (Oxygen production in light phase): Oxygen production measured during the light phase.
- Rd (Oxygen consumption in dark phase): Oxygen consumption measured during the dark phase.

Oxygen data collected under both dark and light conditions were normalized first by cell number and then by chlorophyll a (Chl a) content in the culture. As a result, the oxygen evolution rates (P and Rd) were expressed as (μmol O_2_ (μg Chl a)^−1^ s^−1^) per cell.

Gas exchange measurements were performed in triplicate for each set of experimental conditions. For each trial, the samples were exposed to a 3 min dark period for Rd measurements, followed by a 3 min light exposure for P measurements, with alternating cycles between the two.

#### 2.6.4. Medium Photosynthesis Rate Value

The gas exchange parameters (**P** and **Rd**) were crucial for calculating the photosynthesis rate using the formula proposed by Henley et al. [24]. By using the data obtained, we were able to calculate the photosynthesis rates for the two strains under varying CO_2_ conditions, without directly presenting the intermediate parameters in the text.

(5) $$P_{rate}=P_{max}[\frac{\alpha PFR}{P_{max}+\alpha PFR}]+R_{d}$$

where

- P*_max_* is the maximum photosynthesis rate (μmol O_2_ (μg Chl a)^−1^ s^−1^) under saturating light conditions.
- α is the initial slope of the P vs. PFR curve (photosynthetic photon flux rate), which represents the photosynthetic efficiency at low light intensity
- PFR curve (photosynthetic photon flux rate), which represents the photosynthetic efficiency at low light intensity.
- Rd the oxygen consumption in dark phase

Subsequently, the average photosynthesis rates of the two strains under both CO_2_ conditions were calculated.

At this aim, we indicated the on the i-th day by Pi rate and 6.

(6) $${\bar{P}}_{rats}=\frac{P_{rats}^{1}+\cdots +P_{rats}^{6}}{6}$$

#### 2.6.5. Medium E_k_ Values

Thanks to gas exchange data, it is possible to also analyze Ek. This parameter is the light intensity at which photosynthesis reaches its maximum rate and does not increase with higher light intensities. It represents the point at which the photosynthetic apparatus becomes saturated with light, and any further increase in light intensity does not significantly enhance the rate of photosynthesis. It is a key parameter used to understand the efficiency of photosynthesis in response to varying light conditions.

Formula for E*_k_*:

(7) $$E_{K}=\frac{P_{max}}{\alpha }$$

where

- P*_max_* is the maximum photosynthesis rate (μmol O_2_ (μg Chl a)^−1^ s^−1^) under saturating light conditions.
- α is the initial slope of the P

Subsequently, the average photosynthesis rates of the two strains under both CO_2_ conditions were calculated.

#### 2.6.6. NPE (Net Photosynthetic Efficiency)

This parameter refers to the efficiency with which solar energy is captured and stored in biomass. It is therefore used as an indicator of productivity. To estimate it, we applied the photosynthetic efficiency formula described by De Vree et al. [25] and Carbone et al. [26].

The formula for calculating NPE is as follows:

(8) $$NPE=\frac{\Delta H_{c}(w(t)-w(0))}{A*s*pm*N*e}$$

where

**ΔH_c_** refers to the standard enthalpy of combustion, with a value of 22.5 kJ per gram.

**w(t)** denotes the dry weight of the biomass measured on day *t*.

**w(0)** represents the initial biomass dry weight at time zero.

**A** indicates the surface area of the bioreactor exposed to light, expressed in square meters; in this case, it is 2.75 m^2^.

**s** refers to the number of seconds of light exposure per day, which in this case is 50, 400 s (calculated by multiplying 14 h by 3600 s per hour).

**pm** is the number of moles of photosynthetically active photons per second per square meter (photons mol s^−1^ m^−2^); in this case, it is derived by multiplying the given PAR value (250) by 10^−6^.

**N** stands for Avogadro’s number.

**e** is the approximate energy of a photon with a wavelength of 400 nm, estimated to be around 4 × 10^−22^ kJ.

#### 2.6.7. Rubisco

Proteins were extracted from microalgal biomass collected during the exponential growth phase. Cells were disrupted on ice using a mortar and pestle in an extraction buffer containing 50 mM Tris-HCl (pH 7.5). After homogenization, the samples were centrifuged at 15,000× *g* for 15 min at 4 °C to remove debris. The resulting supernatant was used as the total protein extract. A quantity of 20 μg of protein per sample was loaded onto a 12% SDS-PAGE gel for separation. Following electrophoresis, gels were stained with Coomassie Brilliant Blue R-250 to visualize protein bands. The large subunit (LSU) of Rubisco was identified according to its molecular weight, with reference to a pre-stained protein marker (BLUltra Prestained Protein Ladder^®^, Thailand), as previously described by Tantray [27]. (2020). Gel images were scanned (Brother MFC-J497DW V 5.1) and analyzed using ImageJ software to quantify the LSU band by measuring its area. Results were reported as percentage area ± standard deviation (SD).

### 2.7. Statistical Analysis

Comparative analyses of growth and photosynthesis data between microalgae strains 053 and 054 were performed using an independent two-sample Student’s *t*-test in Microsoft Excel (Analysis ToolPak). Data are presented as mean ± standard deviation (SD), and differences were considered statistically significant when *p* < 0.05.

## 3. Results

### 3.1. Growth Response of Microalgae to Atmospheric vs. Elevated CO_2_ Concentrations

**Figure 1:**
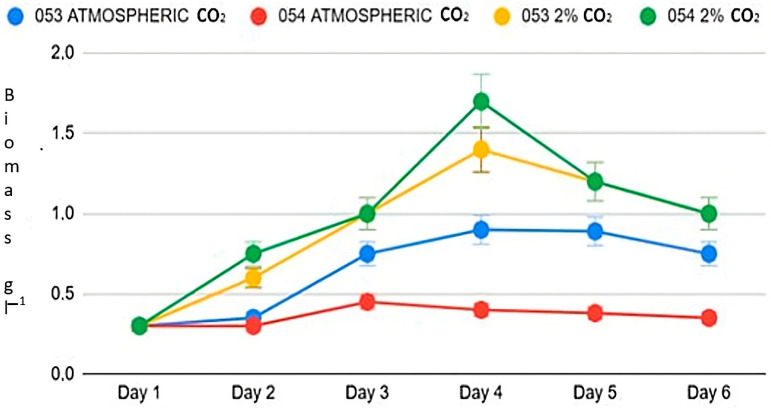
Growth trend of the two strains.

At atmospheric concentrations, algal strains 053 and 054 display distinct growth patterns (Figure 1). Although both start from the same initial algal biomass concentration of 0.3 g L^−1^, strain 053 reaches a maximum growth rate of 0.199 d^−1^ on day three, while strain 054 reaches a significantly lower value of 0.08 d^−1^, which is just under half that of strain 053 (*p* < 0.05). Furthermore, strain 053 achieves its peak biomass concentration on day four, reaching 0.9 g L^−1^, whereas strain 054 reaches its maximum biomass on day three, with 0.4 g L^−1^ (Figure 1).

Exposure to 2% CO_2_ in the atmosphere results in a substantial increase in biomass for both strains. Under these enriched conditions, strain 054 exhibits growth behavior similar to that of strain 053 (*p* < 0.05). Both strains reach their maximum growth rate on day two: strain 053 reaches 0.3 d^−1^, while strain 054 achieves 0.37 d^−1^. Additionally, both strains reach their peak biomass on day four.

These results indicate a significant enhancement in growth relative to ambient conditions without supplemental CO_2_: strain 053 increases its maximum biomass by 66%, while strain 054 demonstrates a 277% increase. In other words, with 2% CO_2_, strain 053 generates about one-third more biomass compared to atmospheric conditions, while strain 054 nearly quadruples its biomass.

### 3.2. pH Values During the Experiment

**Figure 2:**
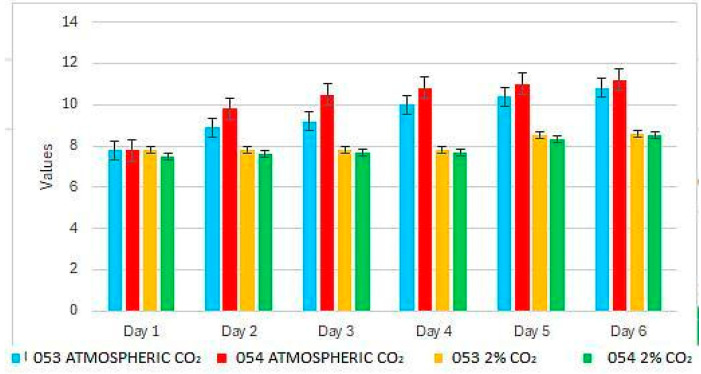
pH variation during the experiment.

At the beginning of the experiment, the pH is uniform across all conditions, with an initial value of 7.8 (Figure 2). By the second day, significant differences are observed between the strains: strain 053 reaches pH values around 9, while strain 054 approaches 10. By the end of the experiment, both strains exhibit a pH of approximately 11.

In treatments with 2% CO_2_, both strains display similar behavior, maintaining pH values that do not exceed 8.5 by the end of the experiment (Figure 2).

### 3.3. Photosynthetic Performance

#### 3.3.1. Characterization of Photochemical Activity

Photochemical activity was measured to assess differences in photosynthetic performance between the two strains.

Under atmospheric CO_2_ conditions, the ΦPSII (Formula (2)) differs markedly between strain 053 and strain 054. On the first day, at 39 μmol photons m^−2^ s^−1^, the value for strain 053 is 0.720, while strain 054 shows a lower efficiency of 0.640 (*p* < 0.05). By day four, under a light intensity of 496 μmol photons m^−2^ s^−1^, strain 053 drops to 0.346, whereas strain 054 declines further to 0.273 (*p* < 0.05). On day six, at 300 μmol photons m^−2^ s^−1^, the value for strain 053 is 0.140, while strain 054 reaches a much lower value of 0.050 (*p* < 0.05).

Under 2% CO_2_ conditions, both strains display similar ΦPSII values during the early stages, with day-one measurements comparable to those of strain 053 under atmospheric CO_2_ (Table S1; *p* > 0.05). However, the decline in ΦPSII is significantly slower for both strains under elevated CO_2_. By day four, at 496 μmol photons m^−2^ s^−1^, both strains show values around 0.300, corresponding to a decrease of approximately 46%. In contrast, under atmospheric CO_2_, strain 053 exhibited a 64% reduction, while strain 054 experienced a 79% decrease over the same period.

Non-Photochemical Quenching (NPQ) also varies with CO_2_ levels and remains consistently lower in strain 053 compared to strain 054 throughout the entire measurement period. For example, on day five, at a light intensity of 89 μmol photons m^−2^ s^−1^, strain 053 shows an NPQ value of 0.098, whereas strain 054 reaches a significantly higher value of 0.480 (Formula (3)).

In the final measurement, conducted at the highest light intensity tested (496 μmol photons m^−2^ s^−1^), the NPQ of strain 054 is higher than that of strain 053.

Under 2% CO_2_, both microalgae exhibit behavior similar to strain 053 under atmospheric conditions, maintaining NPQ values below 0.150 at a light intensity of 39 μmol photons m^−2^ s^−1^. However In the measurements taken on days 5 and 6, the values for strain 054 were observed to be lower than that 053.

**Figure 3:**
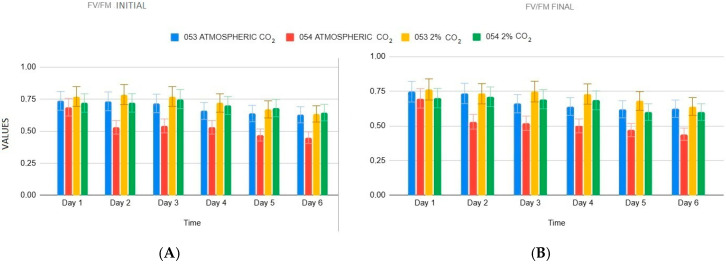
(**A**) initial *F_v_*/F*_m_* values (**B**) final F*_v_*/F*_m_* values.

The analysis of the F*_v_*/F*_m_* ratio (Formula (4)) under atmospheric CO_2_ reveals clear differences between the two strains starting from the second day (*p* < 0.05, Figure 3). Specifically, strain 053 maintains both initial and final values above 0.7, whereas strain 054 remains consistently lower, around 0.5, at both the beginning and end of the experiment. By the last day, the F_v_/F_m_ ratio in strain 053 stabilizes around 0.6, while strain 054 undergoes a sharper decline, reaching values close to 0.4.

Under 2% CO_2_ exposure, both strains initially present similar F_v_/F_m_ values, remaining around 0.7 up to day four (*p* > 0.05). However, strain 054 shows a slight decrease in final F*_v_*/F*_m_* values compared to strain 053.

These findings suggest a differential physiological adaptation of the two strains in response to varying CO_2_ concentrations.

#### 3.3.2. Medium Photosynthetic Rate and E_k_ Values

**Figure 4:**
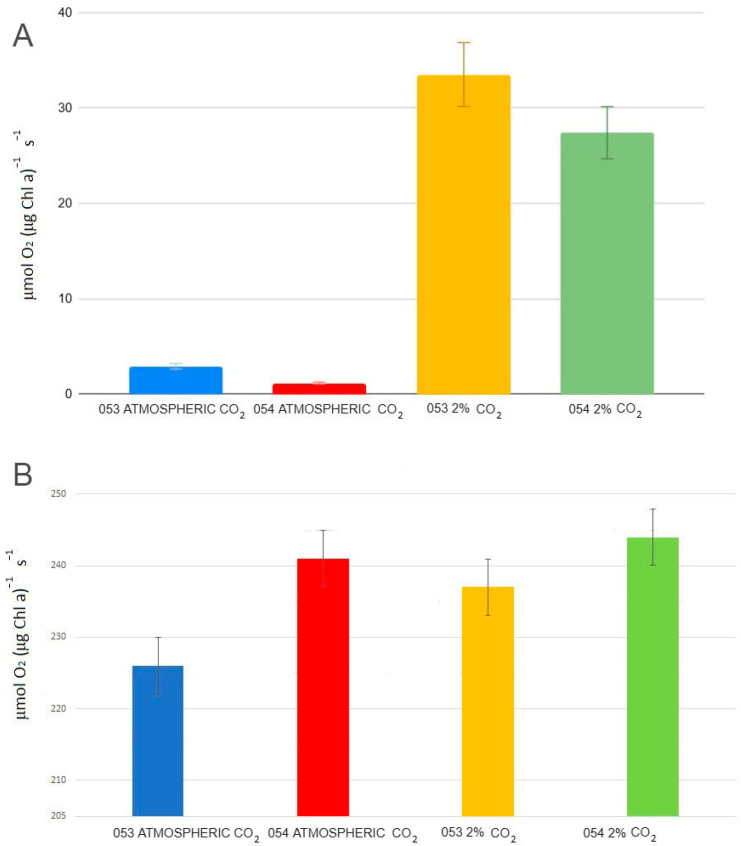
(**A**) Medium photosynthetic rate values (**B**) E*_k_* values.

At ambient CO_2_ concentrations, the average photosynthetic rate (Figure 4; Formula (5)) of strain 053 is more than twice that of strain 054 (1.13 O_2_ (μmol O_2_ (μg Chl a)^−1^ s^−1^) for strain 053 vs. 2.9 (μmol O_2_ (μg Chl a)^−1^ s^−1^) for strain 054) (*p* < 0.05). However, under 2% CO_2_, the photosynthetic rates of both strains converge, becoming similar (*p* < 0.05) (Formulas (5) and (6)). While notable differences are observed in the photosynthetic rates between the two CO_2_ conditions, the E*_k_* values (Formula (7)) for both strains remain comparable across the two CO_2_ levels.

#### 3.3.3. Net Photosynthetic Efficiency

**Figure 5:**
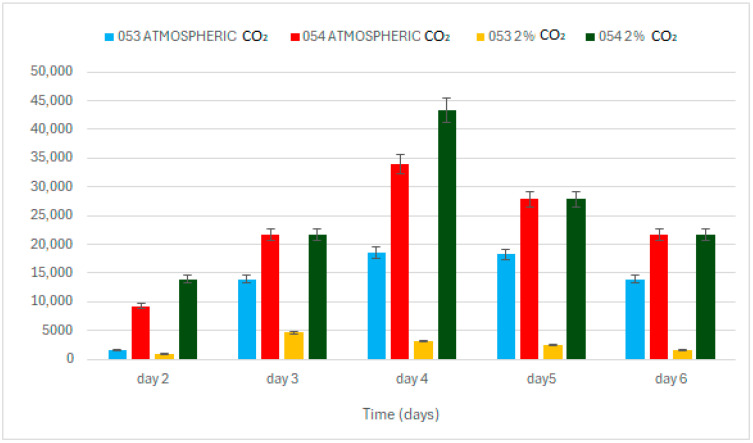
Net photosynthetic efficiency.

Regarding Net Photosynthetic Efficiency (NPE, Formula (7)), the two strains exhibit markedly different responses depending on CO_2_ concentration (*p* < 0.05). Under ambient atmospheric CO_2_ conditions, strain 053 displays NPE values approximately twice as high as those observed in strain 054, indicating a higher photosynthetic efficiency in low-CO_2_ environments. However, under elevated CO_2_ conditions (2%), strain 054 shows a more pronounced increase in photosynthetic efficiency, surpassing the values recorded for strain 053 (Figure 5).

#### 3.3.4. Rubisco Quantification

**Figure 6:**
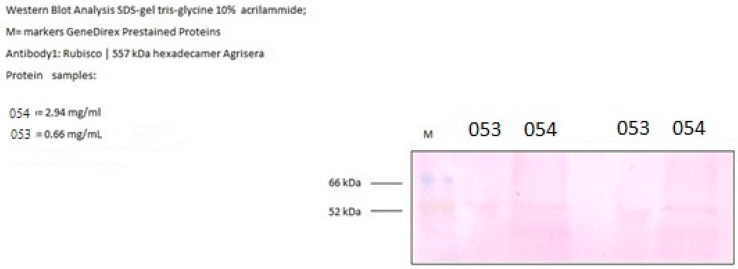
Rubisco quantification.

Rubisco levels were determined by Western blot analysis on samples grown under atmospheric CO_2_ conditions. Quantification showed that strain 054 accumulated 2.94 mg L^−1^ of Rubisco, whereas strain 053 exhibited a lower level of 0.66 mg L^−1^ (Figure 6).

## 4. Discussion

The experiment was conducted at a light intensity of 250 μmol photons m^−2^ s^−1^, as previous studies on the same strains had not revealed any photolimiting phenomena [27,28]. Over six days, the two algal strains were monitored, analyzing the different stages of their development until cell death. From the initial measurements, under atmospheric CO_2_ conditions, significant differences emerged between the two strains: strain 053 exhibited markedly higher growth compared to strain 054, showing both higher biomass and growth rate values. This parameter is a crucial indicator of metabolic efficiency and the ability to proliferate in response to specific environmental factors [29,30].

Strain 054 not only accumulated significantly lower biomass compared to strain 053 but also reached its growth peak one day earlier. This behavior appears to be associated with pH values, which were significantly higher in strain 054 compared to strain 053. pH is indeed one of the most critical environmental parameters in microalgae cultivation, as it directly influences the solubility of gases such as CO_2_ and nutrient availability, thus profoundly affecting algal metabolism and growth [31].

Throughout the growth cycle, pH tends to increase progressively due to the consumption of inorganic carbon by the algae. However, at high pH values, the availability of dissolved CO_2_ decreases, as the gas mainly converts into bicarbonate (HCO_3_^−^), a form that is less readily utilized than free CO_2_. This reduction in dissolved CO_2_ can severely limit cellular growth [32]. When the pH exceeds approximately 10, the growth of both strains is severely compromised. Such pH-dependent inhibition of growth has been observed in other green microalgae as well: for instance, *Chlorella vulgaris* shows reduced growth above pH 10 due to limited CO_2_ uptake while *Scenedesmus obliquus* exhibits decreased photosynthetic efficiency under similar alkaline conditions [33,34]. The fact that strain 054 reached its maximum growth before strain 053 suggests the presence of differences in the optimal pH requirements between the two strains, directly influencing their growth rates.

When the CO_2_ concentration was increased to 2%, the pH of both cultures only slightly increased towards the end of the experiment, reaching similar values. This behavior is explained by the role of dissolved CO_2_ in the carbonic equilibrium: the formation of carbonic acid (H_2_CO_3_) and its subsequent dissociation into H^+^ and HCO_3_^−^ tend to lower the pH. When CO_2_ availability decreases, the concentration of H^+^ ions drops, leading to an increase in pH [35,36,37].

This mechanism has direct effects on growth: with the increase in CO_2_, both strains significantly improved their growth performance, and the differences observed under atmospheric conditions were nullified [38,39,40]. The increase in CO_2_—and its effect on pH—also reflected in parameters related to photosynthetic performance. Specifically, the photochemical activity, which refers to the efficiency with which light energy is converted into chemical energy during photosynthesis, was significantly higher in strain 053 compared to strain 054 under atmospheric CO_2_ conditions.

The parameters F*_v_*/F*_m_* and ΦPSII are commonly used to quantify the photosynthetic efficiency of Photosystem II (PSII). F*_v_*/F*_m_* measures the maximum efficiency of PSII under dark conditions and is considered an indicator of the physiological state of the culture [41], while ΦPSII reflects the efficiency of PSII under lights conditions (photochemical activity).

Under atmospheric CO_2_, strain 053 had an initial F*_v_*/F*_m_* value of about 0.7, indicating excellent photosynthetic efficiency under non-stressful conditions [42], and this value remained optimal throughout the experiment. In contrast, strain 054 showed lower initial values, around 0.6, and a gradual decline, dropping below 0.5, indicating severe photoinhibitory stress and progressively compromised photosynthetic efficiency [38,39], As extensively documented, ΦPSII values tend to decrease with increasing light stress [40,41], For strain 054, this decrease was particularly dramatic, with ΦPSII approaching zero at high light intensities in the final stages.

Under 2% CO_2_, however, ΦPSII values improved in both strains, indicating that increased CO_2_ can mitigate the effects of light stress [42,43], thus improving photosynthetic efficiency even under intense light conditions. This positive effect of elevated CO_2_ was also reflected in the response of NPQ (Non-Photochemical Quenching), a mechanism for dissipating excess light energy. Under atmospheric CO_2_, strain 054 showed very high NPQ values (>1.0) at high light intensities, signaling strong activation of protective mechanisms to prevent oxidative damage [41,44],

However, under 2% CO_2_, the NPQ value decreased in strain 054, indicating a more efficient utilization of incoming light energy and a reduced need for non-photochemical energy dissipation. This optimization of photoprotective mechanisms results in a more effective use of absorbed light energy for photosynthetic processes, ultimately enhancing photosynthetic productivity. This improvement is consistent with the trend observed in Net Photosynthetic Efficiency (NPE) values, which show a parallel increase under the same conditions. The similar behavior of NPQ and NPE suggests a reduction in energy losses and a more efficient conversion of light energy into fixed carbon, highlighting a favorable metabolic adjustment of strain 054 to elevated CO_2_ availability.

In essence, although strain 054 initially showed greater vulnerability, it proved to be more metabolically flexible: in the presence of high CO_2_, it managed light utilization more effectively and reduced oxidative stress compared to strain 053.

To complete the analysis of photosynthesis, photochemical activity data were integrated with gas exchange measurements, specifically analyzing the average P rate and E*_k_* [24,45,46,47]. The average photosynthetic rate (P rate) is higher in strain 053 compared to 054 under atmospheric CO_2_ conditions but becomes equivalent at higher CO_2_ concentrations. In contrast, E*_k_* does not appear to be affected by CO_2_ levels, suggesting a similar response of both strains to light [48,49]. This could be due to the saturation of the photosynthetic capacity or optimal regulation of Rubisco.

Supporting these observations, Rubisco production was also analyzed, revealing an interesting aspect. Despite the higher photosynthetic and gas exchange activity, the amount of Rubisco produced in strain 053 was significantly lower compared to strain 054. This phenomenon appears to be linked to intracellular pH: a lower pH in strain 053 favors the functioning of the CO_2_ Concentrating Mechanism (CCM), which facilitates the transport of bicarbonate and its conversion into CO_2_ near the Rubisco enzyme. This strategy allows strain 053 to optimize photosynthetic efficiency by reducing the amount of Rubisco needed, saving nitrogen resources without compromising photosynthetic performance [50,51].

Understanding these interconnections is key for applying the results to biotechnological cultivation. These results emphasize the critical role of photosynthetic parameters in optimizing microalgal cultivation in photobioreactors. The photochemical activity of PSII governs the conversion of light into ATP and NADPH, which are essential for metabolite synthesis, while a well-regulated NPQ dissipates excess energy and prevents oxidative damage, thereby enhancing overall productivity [52]. Gas exchange parameters, such as Ek and maximum photosynthesis rate, further provide predictive insights into strain productivity. High Ek values indicate the ability to efficiently utilize high light intensities without experiencing photoinhibition, whereas a high maximum photosynthesis rate reflects the capacity to sustain elevated carbon fixation rates, ensuring a continuous supply of ATP, NADPH, and precursors for metabolite synthesis [53]. Together, these features enable strains to maintain an active metabolism under intense light and elevated CO_2_, maximizing biomass accumulation and triglyceride production. Such mechanisms are particularly relevant for lipid synthesis: strains with a high-performing PSII, elevated ΦPSII, and balanced NPQ achieve maximal triglyceride accumulation without compromising cell health, as also demonstrated in previous studies on *Graesiella emersonii* [54,55]. From a biotechnological perspective, a comprehensive understanding of strain photosynthetic performance is essential for translating these findings to large-scale cultivation. This knowledge allows the optimization of production while minimizing inefficiencies and costs associated with photobioreactor operation [55,56]. By carefully adjusting light intensity, CO_2_ levels, and pH, photosynthetic stress can be mitigated, and metabolism directed toward lipid accumulation, providing targeted and sustainable strategies for industrial microalgal cultivation.

## 5. Conclusions

This study highlights that the two strains of *Graesiella emersonii* analyzed, originating from contrasting ecological contexts, exhibit marked differences in their physiological adjustment to CO_2_ supply. Such variability indicates that the ecological history of a strain can influence its carbon assimilation capacity and overall growth dynamics. Rather than considering microalgae as interchangeable biological tools, these findings suggest the need for a strain-specific approach in biotechnological development. Future research should therefore not only validate these responses under larger-scale and fluctuating cultivation conditions but also explore the molecular and metabolic mechanisms underlying the observed differences. Clarifying these aspects could guide the rational selection and engineering of strains capable of sustaining higher productivity and stability, with potential applications in sustainable biomass generation, carbon capture, and bio-based industries.

## Data Availability

Inquiries can be directed to the corresponding author.

## Supplementary Materials

The following supporting information can be downloaded at: https://www.mdpi.com/article/10.3390/bioengineering12101061/s1.
