# Supplementary material for: Adaptation of Graesiella emersonii Strains to Atmospheric and Enriched CO2: Exploring Growth and Photosynthetic Efficiency
Source: Bioengineering (Basel). 2025 Sep 30;12(10):1061. doi: 10.3390/bioengineering12101061 (PMC12561443; doi:10.3390/bioengineering12101061)
Supplement: Supplementary file 1 [file bioengineering-12-01061-s001.zip › bioengineering-3821207-supplementary.pdf]

Supplementary material

S1 The Tubular bioreactor (Tubular-B) consisted of a glass column photobioreactor featuring a light-exposed surface area of 275 cm². A glass tube connected to a membrane pump with a sterile filter at the base provided aeration throughout the system. The reactor's total volume was 350 mL, with a working volume of 200 mL .This design was selected due to its ability to achieve efficient mixing of the culture suspension.

Figure S1:

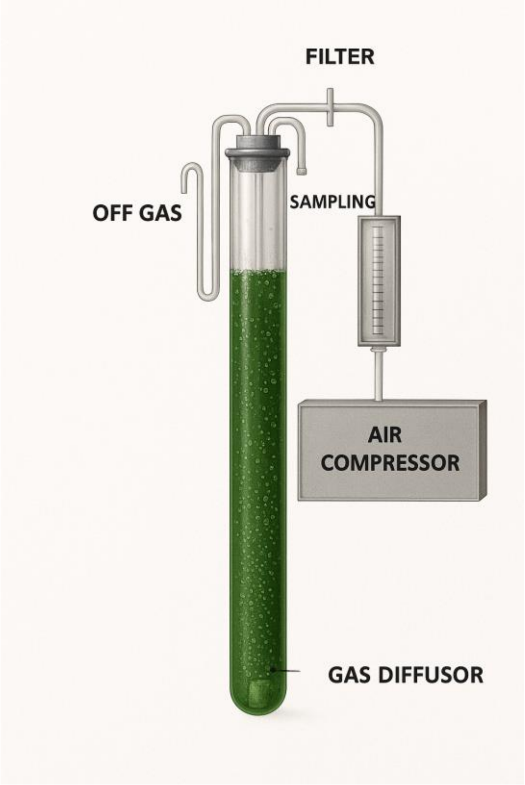

:

1) Table S1 . ΦPSII values of 053 and 054 in presence of different CO2 conditions and at different light intensities.

ΦPSII												
	ATMOSPHERIC CO_2											
	053						054					
**PPFD**	**DAY 1**	**DAY 2**	**DAY 3**	**DAY 4**	**DAY 5**	**DAY 6**	**DAY 1**	**DAY 2**	**DAY 3**	**DAY 4**	**DAY 5**	**DAY 6**
39	*0.720*	*0.644*	*0.584*	*0.500*	*0.485*	*0.400*	*0.640*	*0.520*	*0.479*	*0.431*	*0.300*	*0.200*
Err	*0.001*	*0.002*	*0.001*	*0.002*	*0.003*	*0.004*	*0.002*	*0.001*	*0.003*	*0.001*	*0.002*	*0.001*
89	*0.663*	*0.580*	*0.523*	*0.470*	*0.400*	*0.340*	*0.591*	*0.430*	*0.373*	*0.293*	*0.210*	*0.15*
Err	*0.001*	*0.002*	*0.001*	*0.005*	*0.004*	*0.002*	*0.002*	*0.003*	*0.004*	*0.004*	*0.005*	*0.001*
300	*0.465*	*0.378*	*0.306*	*0.287*	*0.218*	*0.170*	*0.392*	*0.25*	*0.126*	*0.1*	*0.1*	*0.05*

Err	*0.001*	*0.001*	*0.001*	*0.001*	*0.003*	*0.007*	*0.004*	*0.002*	*0.001*	*0.003*	*0.004*	*0.005*
496	*0.346*	*0.255*	*0.209*	*0.177*	*0.140*	*0.090*	*0.23*	*0.18*	*0.095*	*0.088*	*0.05*	*0.000*
Err	*0.001*	*0.002*	*0.001*	*0.002*	*0.003*	*0.004*	*0.002*	*0.001*	*0.003*	*0.001*	*0.002*	*0.001*
								**2% CO_2**				
				**053**							**054**	
39	*0.754*	*0.714*	*0.695*	*0.6*	*0.566*	*0.468*	*0.72*	*0.68*	*0.668*	*0.61*	*0.52*	*0.441*
Err	*0.001*	*0.002*	*0.001*	*0.002*	*0.003*	*0.004*	*0.002*	*0.001*	*0.003*	*0.001*	*0.002*	*0.001*
89	*0.691*	*0.661*	*0.593*	*0.55*	*0.403*	*0.384*	*0.65*	*0.6*	*0.561*	*0.4*	*0.4*	*0.394*
Err	*0.001*	*0.002*	*0.001*	*0.002*	*0.003*	*0.004*	*0.002*	*0.001*	*0.003*	*0.001*	*0.002*	*0.001*
300	*0.57*	*0.5*	*0.42*	*0.37*	*0.333*	*0.233*	*0.54*	*0.483*	*0.482*	*0.288*	*0.35*	*0.185*
Err	*0.001*	*0.002*	*0.001*	*0.002*	*0.003*	*0.004*	*0.002*	*0.001*	*0.003*	*0.001*	*0.002*	*0.001*
496	*0.5*	*0.4*	*0.37*	*0.27*	*0.24*	*0.142*	*0.49*	*0.42*	*0.39*	*0.28*	*0.206*	*0.135*
Err	*0.001*	*0.001*	*0.001*	*0.001*	*0.003*	*0.007*	*0.004*	*0.002*	*0.001*	*0.003*	*0.004*	*0.005*

2) Table S2. NPQ values of 053 and 054 in presence of different CO2 conditions and at different light intensities

							NPQ					
								**ATMOSPHERIC CO_2**				
				**053**							**054**	
**PPFD**	**DAY 1**	**DAY 2**	**DAY 3**	**DAY 4**	**DAY 5**	**DAY 6**	**DAY 1**	**DAY 2**	**DAY 3**	**DAY 4**	**DAY 5**	**DAY 6**
39	*0.034*	*0.038*	*0.048*	*0.025*	*0.036*	*0.090*	*0.070*	*0.072*	*0.052*	*0.110*	*0.115*	*0.14*
Err	*0.0001*	*0.0002*	*0.0001*	*0.0002*	*0.0003*	*0.0004*	*0.002*	*0.001*	*0.003*	*0.001*	*0.002*	*0.001*
89	*0.099*	*0.076*	*0.198*	*0.132*	*0.098*	*0.180*	*0.165*	*0.210*	*0.220*	*0.360*	*0.48*	*0.5*
Err	*0.0001*	*0.002*	*0.001*	*0.005*	*0.0004*	*0.002*	*0.002*	*0.003*	*0.004*	*0.004*	*0.005*	*0.001*
300	*0.186*	*0.278*	*0.378*	*0.478*	*0.621*	*0.700*	*0.430*	*0.499*	*0.533*	*0.723*	*0.8*	*0.93*
Err	*0.001*	*0.001*	*0.001*	*0.0001*	*0.003*	*0.007*	*0.004*	*0.002*	*0.001*	*0.003*	*0.004*	*0.005*
496	*0.259*	*0.420*	*0.596*	*0.735*	*0.880*	*0.920*	*0.590*	*0.68*	*0.723*	*0.9*	1,0	1,823
Err	*0.001*	*0.002*	*0.001*	*0.0002*	*0.003*	*0.004*	*0.002*	*0.001*	*0.003*	*0.001*	*0.002*	*0.001*
								**2% CO_2**				
					**053**						**054**	
39	*0.041*	*0.038*	*0.048*	*0.05*	*0.04*	*0.1*	*0.011*	*0.032*	*0.028*	*0.04*	*0.04*	*0.123*
Err	*0.001*	*0.002*	*0.001*	*0.002*	*0.003*	*0.004*	*0.002*	*0.001*	*0.003*	*0.001*	*0.002*	*0.001*

89	0.098	0.085	0.128	0.18	0.203	0.2	0.053	0.073	0.080	0.080	0.19	0.210
Err	*0.001*	*0.002*	*0.001*	*0.002*	*0.003*	*0.004*	*0.002*	*0.001*	*0.003*	*0.001*	*0.002*	*0.001*
300	0.204	0.3	0.42	0.5	0.7	0.8	0.152	0.302	0.282	0.288	0.6	0.65
Err	*0.001*	*0.002*	*0.001*	*0.002*	*0.003*	*0.004*	*0.002*	*0.001*	*0.003*	*0.001*	*0.002*	*0.001*
496	0.289	0.414	0.52	0.72	0.82	0.9	0.242	0.44	0.42	0.5	0.7	0.798
Err	*0.001*	*0.001*	*0.001*	*0.001*	*0.003*	*0.007*	*0.004*	*0.002*	*0.001*	*0.003*	*0.004*	*0.005*
